# Kinetic arrest of the ferromagnetic state in Mn$_3$GaC and Ni$_2$MnGa composite mixtures

**DOI:** 10.1038/s41598-021-99005-5

**Published:** 2021-10-01

**Authors:** K. R. Priolkar, R. Nevgi, E. T. Dias, A. K. Nigam

**Affiliations:** 1 School of Physical and Applied Sciences, Goa University, Taleigao Plateau, Goa, 403206 India; 2 Tata Institute of Fundamental Research, Dr. Homi Bhabha Road, Colaba, Mumbai, 400005 India

**Keywords:** Condensed-matter physics, Magnetic properties and materials

## Abstract

The kinetics of the ferromagnetic to antiferromagnetic transition in Mn$_3$GaC can be arrested and its magnetic properties can be tuned by mixing a small amount ($\sim$ 10%) of Heusler Ni$_2$MnGa to Mn$_3$GaC. A detailed study of magnetic properties of composite mixtures of Mn$_3$GaC and Ni$_2$MnGa with different antiperovskite to Heusler ratio, reveals that the ferromagnetic Ni$_2$MnGa polarizes magnetic spins of the antiperovskite phase by creating a magnetic strain field in its vicinity. The Heusler phase acts as a defect centre whose influence on the magnetic properties of the majority antiperovskite phase progressively diminishes, creating a distribution of transition temperatures. Such strong interaction between the two phases of the mixture allows for tunability and control over the properties of such magneto-structurally transforming materials.

## Introduction

Phase separation or phase co-existence is a general feature of materials exhibiting first-order phase transition and is associated with superheating or supercooling. A plethora of studies have shown the possibility of modulating/arresting the kinetics of phase transition by altering the thermodynamic conditions as the material undergoes a transition from one phase to another^1^. Phase separation plays a key role in the magnetic and magnetocaloric properties of Mn-based antiperovskites^2^. Novel phenomena like shell ferromagnetism in Ni$_{50}$Mn$_{45}$In$_5$ is also ascribed to its phase separation into antiferromagnetic Ni$_{50}$Mn$_{50}$ and a ferromagnetic Ni$_{50}$Mn$_{25}$In$_{25}$ activated by thermal annealing^3,4^. Phase separation and shell ferromagnetism have strong implications on the magnetocaloric effect in Ni–Co–Mn–In based Heusler compounds^5^. The appearance of strain glassy ground state in Fe doped Ni–Mn–In based Heusler alloys is linked to phase separation^6^. Further, magnetic phase separation leading to a non-ergodic ground state occurs in Mn$_3$Ga$_{0.45}$Sn$_{0.55}$C. Here the material appears crystallographically single phase but consists of Ga-rich and Sn-rich clusters. These Ga-rich and Sn-rich clusters order antiferromagnetically with the same propagation vector as Mn$_3$GaC and Mn$_3$SnC respectively, and the interaction between them renders the material to exhibit a cluster glassy ground state^7^. Exchange bias and kinetic arrest are the two other phenomena that have been observed in magnetically phase-separated compounds^8–14^. In a compound with competing ferromagnetic and antiferromagnetic interactions, the ferromagnetic entities gain in strength when cooled from the high-temperature paramagnetic state through its magnetostructural transition under the influence of the magnetic field. However, the crystallographic single-phase nature of the compound makes it difficult to understand the magnetic coupling between the two phases.

The antiperovskite, Mn$_3$GaC transitions to a high volume antiferromagnetic ground state via a first-order magnetostructural transition from a low volume cubic ferromagnetic state at $T_{ms} \sim 170$ K^15,16^. This magnetostructural transition in Mn$_3$GaC can be reversed under the influence of external magnetic field^17^. Even at temperatures as low as 5 K, a magnetic field of 20 T is sufficient to induce the ferromagnetic state^18^. A small decrease in the magnetic field reverts the compound to its antiferromagnetic state implying very weak kinetic arrest. However, cooling a carbon deficient Mn$_3$GaC$_x$ ($x \sim 0.8$) in a magnetic field of 5 T down to 5 K results in an arrest of the high-temperature ferromagnetic state^19^. Inhomogeneous magnetic ground state with kinetic arrest features has recently been reported in Mn$_{3-x}$Ni$_x$GaC^20^. Addition of Ni leads to a formation of a multiphasic compound comprising of a Heusler impurity along with the major antiperovskite phase. Magnetically frustrated ground states with glassy dynamics due to structural phase separation into a major antiperovskite phase and a minor Heusler impurity phase has been reported in Mn$_{2.8}$T$_{0.2}$SnC (T = Co, Ni and Cu)^21^.

Though structural phase separation appears to play a key role in the kinetics of the phase transition as well as the ground state of the above antiperovskites, the minor phase fraction of the impurity phase makes it difficult to conclude the same. Is it possible then to replicate such properties by preparing mixtures or composites of the two phase-separated members? Such an approach may also enable a better control in tailoring their properties as well as in the introduction of new materials in the future. We propose to test this hypothesis by preparing mixtures of Mn$_3$GaC and Ni$_2$MnGa and observing the kinetic arrest phenomena seen in Mn$_{3-x}$Ni$_x$GaC. We have prepared three mixtures of ($1-y$) [Mn$_3$GaC] + *y* [Ni$_2$MnGa] (*y* = 0.05, 0.1 and 0.25). Although the structural features of the antiperovskite and Heusler components are visible in the composite mixtures, their magnetic properties differ greatly from the two end members. Interestingly the phenomenon of kinetic arrest reported in Mn$_{2.9}$Ni$_{0.1}$GaC is visible in 0.9 [Mn$_3$GaC] + 0.1 [Ni$_2$MnGa].

## Results

**Figure 1:**
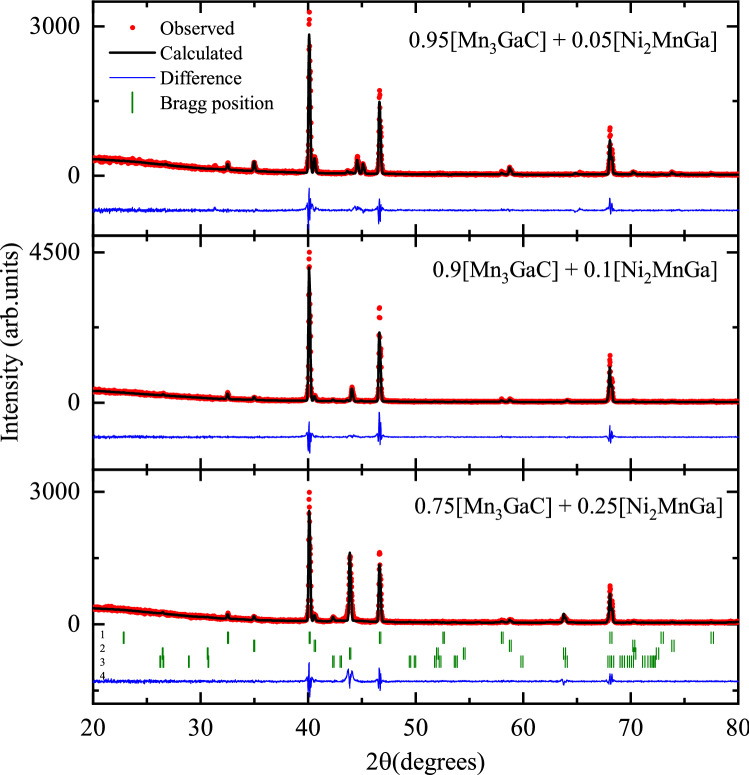
Rietveld refined X-ray diffraction patterns of Mn$_3$GaC and Ni$_2$MnGa composites. The four identified phases are 1. Mn$_3$GaC, 2. MnO, 3. Ni$_2$MnGa cubic and 4. Ni$_2$MnGa monoclinic.

**Figure 2:**
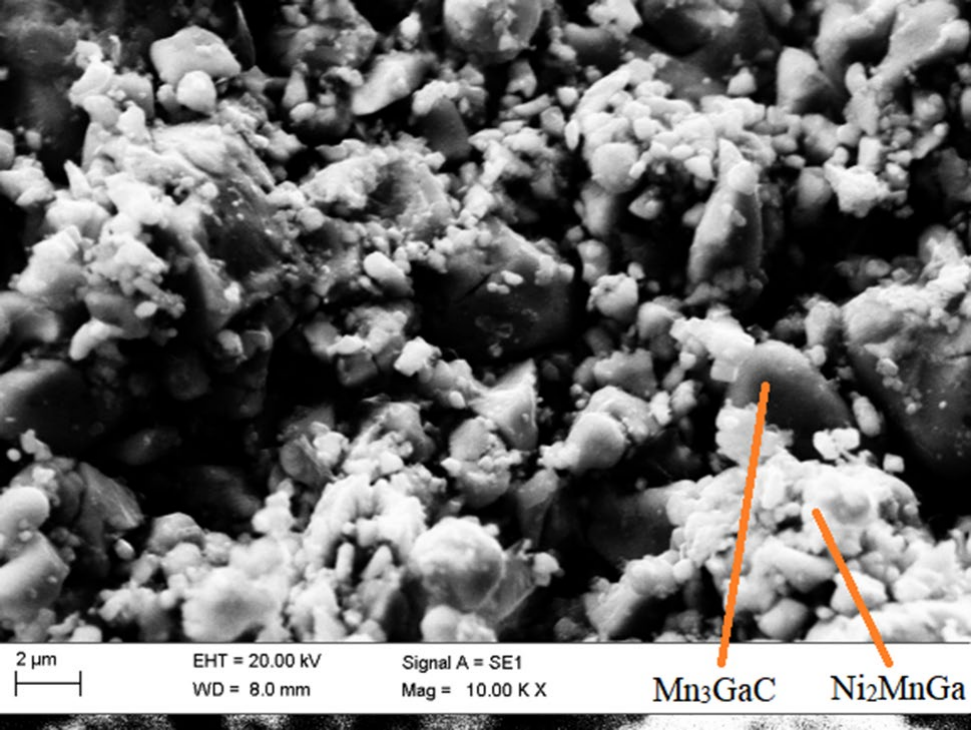
SEM micrograph showing the two phases in 0.75 [Mn$_3$GaC] + 0.25 [Ni$_2$MnGa].

Rietveld refined X-ray diffraction patterns of the three composite mixtures of ($1-y$) [Mn$_3$GaC] + *y* [Ni$_2$MnGa] (*y* = 0.05, 0.1 and 0.25) are presented in Fig. 1. The presence of Bragg peaks corresponding to antiperovskite and Heusler phases confirms the formation of composites. Four structural phases, Mn$_3$GaC ($Pm{\bar{3}}m$), cubic (austenitic) Ni$_2$MnGa ($Fm{\bar{3}}m$), monoclinic (martensitic) Ni$_2$MnGa ($P2_1$) and MnO ($Fm{\bar{3}}m$), were used to fit the diffraction patterns of the composites. For the monoclinic phase, structural parameters reported by Righi et al.^22^ were used. The percentage of monoclinic phase is small (maximum $\sim$ 3%) and cannot be clearly seen except in the composite sample with *y* = 0.05. Martensitic transition temperature of Ni$_2$MnGa critically depends on the alloy composition. Small variation especially increase in Ni content at the expense of Mn raises the transition temperature around room temperature. Presence of MnO impurity phase can alter the Ni to Mn ratio in the Heusler phase which can be the reason for the presence of monoclinic phase in the diffraction patterns. The refined values of lattice constants of the constituent phases as well as their phase fractions obtained from the refinement are reported in Table 1. The values of lattice constants of Mn$_3$GaC and Ni$_2$MnGa in the three composites agree well with the reported values of 3.89 Å^16^ and 5.825 Å^23^ respectively. Presence of antiperovskite and Heusler phases was also verified from SEM micrographs and SEM-EDX spectroscopy and can be seen for one representative composite sample in Fig. 2.

**Table 1:** The refined crystallographic data for the composites ($1-y$) [Mn$_3$GaC] + *y* [Ni$_2$MnGa] with *y* = 0.075, 0.1 and 0.2.

Composite	Phase name (space group)	Phase %	Lattice parameters
0.95 [Mn$_3$GaC]	Mn$_3$GaC ($Pm{\bar{3}}m$)	81.7(8)	*a* = 3.8932(1)
+	MnO ($Fm{\bar{3}}m$)	12.83	*a* = 4.4428(1)
0.05 [Ni$_2$MnGa]	Ni$_2$MnGa ($Fm{\bar{3}}m$)	5.5(2)	*a* = 5.7481(3)
	Ni$_2$MnGa (*I*2/*m*)		*a* = 4.2389(5) *b* = 5.589(1)
			*c* = 4.1511(8) $\beta$ = 92.86(1)
0.9 [Mn$_3$GaC]	Mn$_3$GaC ($Pm{\bar{3}}m$)	89.5(7)	*a* = 3.8939(1)
+	MnO ($Fm{\bar{3}}m$)	4.73	*a* = 4.4434(1)
0.1 [Ni$_2$MnGa]	Ni$_2$MnGa ($Fm{\bar{3}}m$)	5.8(2)	*a* = 5.8097(2)
	Ni$_2$MnGa (*I*2/*m*)		*a* = 4.2788(4) *b* = 5.594(4)
			*c* = 4.213(1) $\beta$ = 93.42(1)
0.75 [Mn$_3$GaC]	Mn$_3$GaC ($Pm{\bar{3}}m$)	66.9(6)	*a* = 3.8930(1)
+	MnO ($Fm{\bar{3}}m$)	5.32	*a* = 4.4439(1)
0.25 [Ni$_2$MnGa]	Ni$_2$MnGa ($Fm{\bar{3}}m$)	27.8(4)	*a* = 5.8336(1)
	Ni$_2$MnGa (*I*2/*m*)		*a* = 4.2788(4) *b* = 5.594(4)
			*c* = 4.213(1) $\beta$ = 93.42(1)

**Figure 3:**
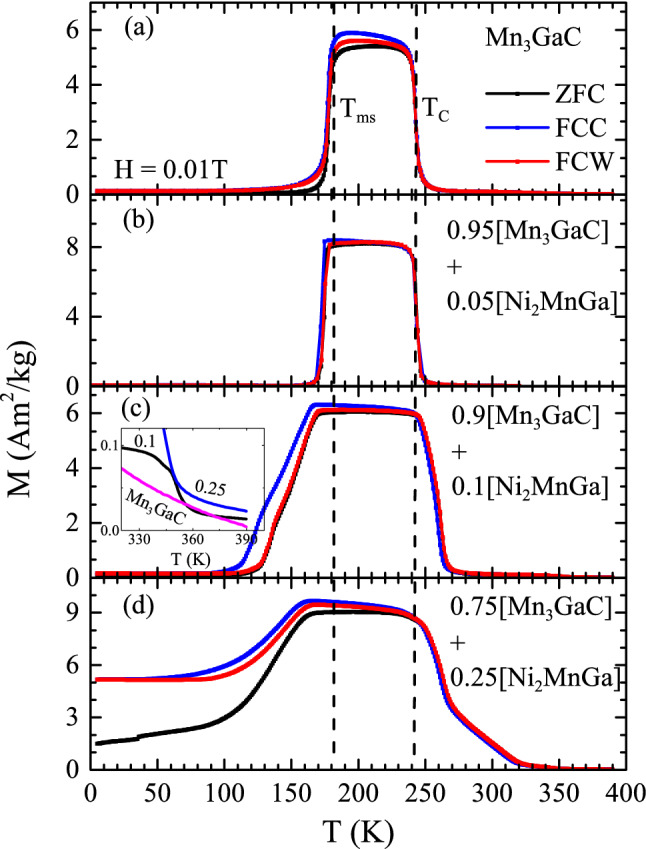
Magnetization as a function of temperature M(T) in Mn$_3$GaC and composites of Mn$_3$GaC and Ni$_2$MnGa with *y* = 0.05, 0.1 and 0.25. The vertical lines indicate positions of $T_{ms}$ and $T_C$ in Mn$_3$GaC. Inset shows expanded view of ZFC magnetization curves in Mn$_3$GaC, *y* = 0.1 and 0.25 in the temperature range 325 K $\le T \le$ 395 K.

Mn$_3$GaC undergoes a paramagnetic to ferromagnetic transition at $T_C =$ 240 K followed by a magnetostructural transition, from low volume cubic ferromagnetic state to high volume cubic antiferromagnetic state at $T_{ms} =$ 175 K^16^. On the other hand, the martensitic transition temperature of Ni$_2$MnGa is $\sim$ 200 K but critically depends on its stoichiometry and the ferromagnetic transition temperature, $T_C$ is around 380 K^24,25^. If these two components of the powder mixture prepared here do not interact with each other, the transition temperatures of the antiperovskite and Heusler phase should remain nearly unchanged in the mixtures. As seen in Fig. 3a–d, both, the $T_C$ and $T_{ms}$ of the antiperovskite Mn$_3$GaC progressively change with increasing Heusler addition. While the ferromagnetism strengthens, as indicated by higher and higher $T_C$, $T_{ms}$ shifts to lower temperatures accompanied by weakening of the antiferromagnetic state. For two of the mixtures, *y* = 0.1 and 0.25, magnetization was recorded up to 390 K to check for the presence of ferromagnetic transition of Ni$_2$MnGa. A weak ferromagnetic transition is visible just below *T* = 350 K (see inset in Fig. 3c), which can be ascribed to the ferromagnetic transition of Ni$_2$MnGa. Further, in the case of composites with low Heusler content (*y* = 0.05 and 0.1) the magnetization value increases systematically with the concentration of the Heusler component. The ZFC magnetization value increases from 0.02 Am$^2$/kg for pure Mn$_3$GaC to 0.05 Am$^2$/kg and 0.1 Am$^2$/kg for the composites with 5% and 10% Heusler content, respectively. This increase in magnetization value with Heusler content, especially in low applied field, can be attributed to the ferromagnetic Ni$_2$MnGa. However, Fig. 3 shows more than an order of magnitude increase in the magnetization value in case of composite with 25% Heusler component. This increase cannot be due to increase in concentration of Heusler component alone.

Another noticeable difference in the composites is the increasing thermal hysteresis between the FCC and FCW curves coupled with the broadening of the magnetostructural transition. Mn$_3$GaC is characterized by a sharp transition and small transformation hysteresis ($\sim$ 1 K) (see Fig. 3a) at its $T_{ms}$. With the addition of the Heusler component, the width of the transition and the transformation hysteresis increases to about 10 K in 0.75 [Mn$_3$GaC] + 0.25 [Ni$_2$MnGa] powder mixture. The broadening of the transition usually refers to a distribution of magnetostructural transition temperatures due to formation of defects. The defect caused here is the inclusion of the minority Heusler phase along with the antiperovskite grains. The ferromagnetic alignment of the Heusler phase influences the alignment of magnetic spins in the antiperovskite phase in its vicinity and thus shifting the ferromagnetic to antiferromagnetic transformation of the antiperovskite phase to a lower temperature. Hence the magnetostructural transition increasingly broadens with the increasing fraction of the Heusler phase. Further, the ZFC magnetization value also progressively increases with the Heusler fraction. In the mixture with *y* = 0.25, the ZFC magnetization value at 5 K is at least an order of magnitude higher in comparison than the same in other mixtures. It is also accompanied by a large splitting between the ZFC and FC magnetization curves. This magnetization behavior can also be related to the presence of the Heusler phase. It may be noted that Ni$_2$MnGa also displays such large splitting in ZFC and FC magnetization curves in its martensitic state^24^. Though the martensitic transition of Ni$_2$MnGa is not explicitly visible in magnetization curves of the composite mixtures, the splitting between ZFC and FC magnetization curves could be related to it.

**Figure 4:**
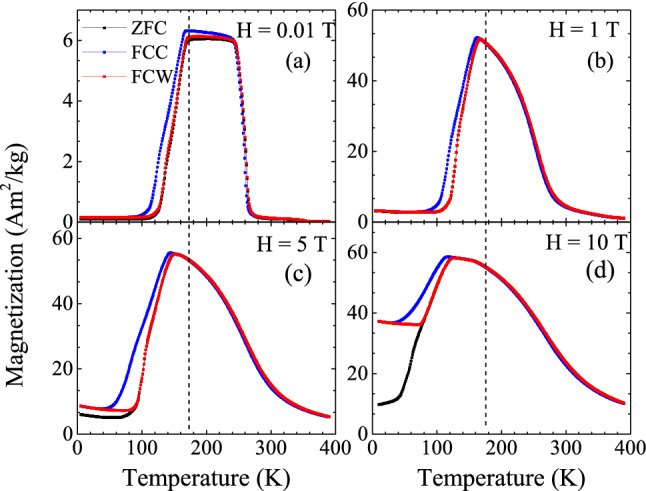
Magnetization as a function of temperature in 0.9 [Mn$_3$GaC] + 0.1 [Ni$_2$MnGa] recorded in different applied fields (**a**) 0.01 T, (**b**) 1 T, (**c**) 5 T and (**d**) 10 T. The dashed vertical line indicates the position of $T_{ms}$ at 0.01 T.

Figure 4a–d presents M(T) measured for 0.9[Mn_3_GaC] + 0.1[Ni_2_MnGa] in fields of 0.01 T, 1 T, 5 T and 10 T respectively during the ZFC, FCC and FCW cycles. With the increase in the magnetic field, though the general features of antiferromagnetic and ferromagnetic transitions remain nearly the same, a splitting between ZFC and FC components is observed. Along with this, the transition hysteresis broadens and the $T_{ms}$ shifts to lower temperature at a rate of $\sim$ 4.9 K/T with increasing applied field. Presence of such features is also noted in magnetically inhomogeneous systems like Mn$_3$GaC$_{0.8}$^19^. The external magnetic field aids the ferromagnetic polarization of antiperovskite phase caused by the Heusler phase which shifts the $T_{ms}$ to a lower temperature. This coupling between the Heusler and antiperovskite modifies the magnetization of antiperovskite and contributes to the increase in magnetization noticed in the ZFC magnetization values at 5 K in Fig. 4. Here, it can be seen that ZFC magnetization value continuously increases with increase in magnetization up to 10 T. This is in contrast to the behavior of Ni$_2$MnGa, which displays a saturating magnetization behavior for H $\ge$ 3 T^26^.

**Figure 5:**
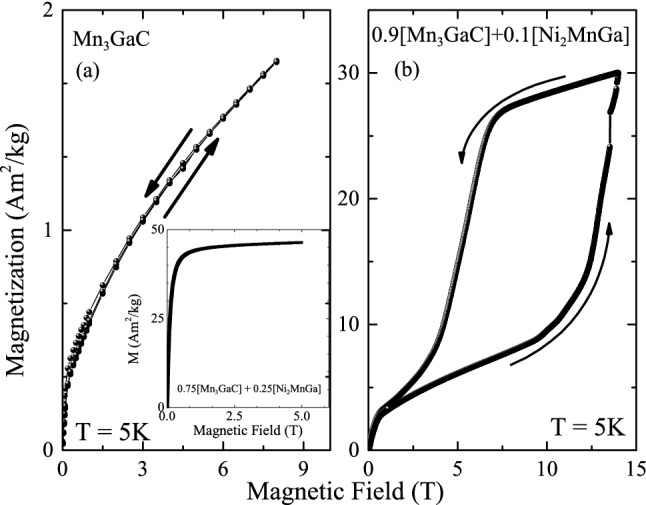
(**a**) Isothermal magnetization curve for Mn$_3$GaC measured during the two cycles of applied magnetic field ($0 T \le H \le 8 T$). Inset shows M(H) loop at 5 K for the first two cycles in 0.75 [Mn$_3$GaC] + 0.25 [Ni$_2$MnGa]. (**b**) Isothermal magnetization curve recorded at T = 5 K for 0.9 [Mn$_3$GaC] + 0.1 [Ni$_2$MnGa] during increasing and decreasing magnetic field from 0 T to +14 T.

To further understand the magnetic coupling between the two components, we examine the field dependence of magnetization M(H) at 5 K. Mn$_3$GaC is antiferromagnetic and the same is reflected in the behavior of magnetization with increasing and decreasing field shown in Fig. 5a. There is no hysteresis, and the magnitude of magnetization is quite low. It may be noted that in Mn$_3$GaC, the low volume ferromagnetic state can be induced even at 5 K with the help of an external magnetic field of 20 T. Addition of ferromagnetic Ni$_2$MnGa to Mn$_3$GaC to realize 0.9 [Mn$_3$GaC] + 0.1 [Ni$_2$MnGa] composite completely changes the character of M(H) curve as can be seen in Fig. 5b. Just above 9 T, one can see a rapid increase in magnetization with its value increasing more than three times signalling a field-induced ferromagnetic state. A reduction of the applied field from 14 T retains this ferromagnetic state down to about 5 T after which the magnetization rapidly decreases with decreasing magnetic field. This decrease in the value of applied field required to induce ferromagnetism at 5 K again supports the above hypothesis of strong interaction between the two components of the mixture, especially in the vicinity of the Heusler antiperovskite phase boundary. With further increase of the Heusler component to 25%, converts the hysteresis loop like that of a soft ferromagnet (see inset in Fig. 5a).

**Figure 6:**
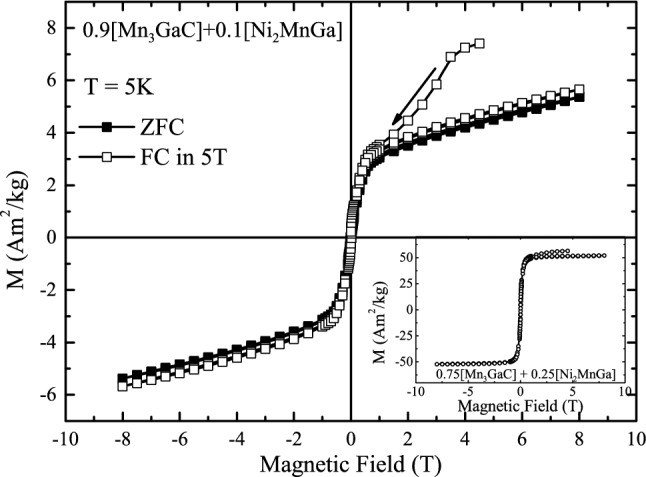
Field cooled (open squares) and zero field cooled (filled squares) hysteresis loop recorded at 5 K in 0.9 [Mn$_3$GaC] + 0.1 [Ni$_2$MnGa]. Inset shows FC hysteresis loops in 0.75 [Mn$_3$GaC] + 0.25 [Ni$_2$MnGa].

Finally, the behavior of M(H) at 5 K when the mixture, 0.9 [Mn$_3$GaC] + 0.1 [Ni$_2$MnGa] is cooled in zero-field (ZFC) and in a fixed applied field (FC) is discussed. The two hysteresis loops are presented in Fig. 6. First, the mixture-sample is cooled in zero-field from 350 to 5 K, and then M(H) is recorded, giving a loop shown by filled squares in the Fig. 6. The magnetization displays a linear increase with the increasing field but bends towards the ordinate axis at around 0.5 T, indicating that presence of ferromagnetic components. Nevertheless, the absence of saturation even at 8 T magnetic field implies the presence of antiferromagnetic components. The hysteresis loop is symmetric around the origin with a very low coercive field ($H_c$ = 0.0125 T) and a magnetization value of ± 5.4 Am$^2$ kg$^{-1}$ at 8 T. The remanence is also negligible at about 0.18 Am$^2$ kg$^{-1}$.

The FC M(H) at 5 K is quite different and is shown with an open square in Fig. 6. Here the sample is cooled to 5 K under an applied field of 5 T from 350 K, and M(H) is measured with field first decreasing to 0 T and then to $-8$ T and back again to $+8$ T. At the start, the magnetization value is 7.4 Am$^2$ kg$^{-1}$ at 5 T. With the reduction in the magnetic field, M(H) decreases rapidly at first and then traces a similar path as the ZFC loop. The remanent magnetization and the coercive field values are also nearly the same as those found in ZFC case. When the field is reversed, the value of magnetization at the maximum negative field (− 8 T) is – 5.4 Am$^2$ kg$^{-1}$, which is smaller in magnitude than the initial value obtained at the start of the loop at 5 T. When the field is reversed again and brought back to 5 T, the magnetization does not acquire the initial value but ends with a value which is about a 50% lower. The maximum value of magnetization obtained at the start of the loop (7.4 Am$^2$ kg$^{-1}$ at 5 T) is not obtained even when the field is increased to 8 T. The magnetization value at 8 T is slightly smaller than that obtained for the ZFC hysteresis loop indicating field-induced ferromagnetic character in the mixture. Such open hysteresis loops indicate partial locking of the ferromagnetic components in the presence of an external magnetic field. They have been reported in magnetically heterogeneous antiperovskites^19,20^ as well as other magnetically phase-separated systems. Open hysteresis loop under field cooling is also observed in *y* = 0.25 mixture even though the ferromagnetic character is more dominant in the hysteresis loop (see inset of Fig. 6).

## Discussion

Field induced ferromagnetism is observed in Mn$_3$GaC^16^. The high volume antiferromagnetic state of Mn$_3$GaC, at temperature below its magnetostructural transition temperature can be reversed to low volume ferromagnetic phase under the application of magnetic field^17^. Removal of magnetic field returns the material back to high volume antiferromagnetic phase and is true even if the sample is cooled in a magnetic field through its transition temperature. However, in carbon deficient Mn$_3$GaC$_{0.8}$, cooling the sample in magnetic field results in a ferromagnetic state instead of antiferromagnetic ground state^19^. The ferromagnetic state stays arrested until the material is heated above its transition temperature. A similar behavior is also seen in Mn$_{3-x}$Ni$_x$GaC. These compounds phase separate into antiperovskite (deficient in Ni) and Ni-rich Heusler phases. The phenomena of kinetic arrest is ascribed to the presence of a ferromagnetic Heusler impurity phase that modifies the properties of the major antiferromagnetic antiperovskite phase.

The magnetic properties of the composite mixtures are similar to that observed in Mn$_{3-x}$Ni$_x$GaC compounds. The strong magnetic coupling between the antiperovskite and the Heusler phase helps in inducing the metamagnetic transition at a much lower value of magnetic field. A broad hysteretic behavior is also noted compared to the pure antiperovskite. Moreover, the ferromagnetic transition of the antiperovskite in 0.9 [Mn$_3$GaC] + 0.1 [Ni$_2$MnGa] composite can be arrested when it is cooled under influence of external magnetic field. A further increase of Heusler content results in a hysteresis characteristic of a soft ferromagnet. Thus providing a useful handle for tuning the field induced ferromagnetism and controlling the hysteresis in Mn$_3$GaC via antiperovskite-Heulser composites.

In composite mixtures, the minor Heusler phase acts as defect centers that modulate the antiferromagnetic order of the the Mn$_3$GaC antiperovskite phase. Though the two components of the composite preserve their structural identities much the same as in their pure form, the ferromagnetic Heusler phase creates a magnetic strain field that polarizes the magnetic alignment of the antiperovskite phase in its vicinity. This polarization lowers the FM to AFM transition temperature of the antiperovskite phase, especially in the phase boundary regions around the Heusler impurity. The influence of the defect on the magnetic properties of the majority antiperovskite phase progressively diminishes as one goes away from these defect centers and thus creates a distribution of transition temperatures. The broadening of the magnetostructural transition with increasing percentage of Heusler phase seen in the magnetization curves as well as the shift in $T_{ms}$ with increasing magnetic field is a direct consequence of the influence of the minority Heusler phase on the magnetism of the majority antiperovskite phase. The polarization also results in an arrest of kinetics of the ferromagnetic to antiferromagnetic transition of the Mn$_3$GaC antiperovskite and results in the open hysteresis loop when cooled under magnetic field and ferromagnetic character down to 5 K.

Though the observed magnetic behavior in the present composite mixtures of Ni$_2$MnGa and Mn$_3$GaC is similar to the phase-separated Mn$_{3-x}$Ni$_x$GaC compounds, the scalability of properties paves way for precise control and tailoring of magnetic properties through the phenomena of kinetic arrest in composite mixtures. Further, the present study opens up a new direction of preparing such composite mixtures and identifying structural and/or magnetic defects that are responsible for the kinetic arrest of magnetostructural transitions in different compounds.

## Methods

The synthesis of composite mixtures of ($1-y$) [Mn$_3$GaC] + *y* [Ni$_2$MnGa] (*y* = 0.05, 0.1 and 0.25) first involved the preparation of the antiperovskite Mn$_3$GaC and the Heusler, Ni$_2$MnGa. Mn$_3$GaC was prepared using the same procedure reported in Ref.^16^. For this, stoichiometric proportions of high purity (> 99.99%) elemental Mn and C powders were carefully mixed with ingots of Ga, pelletized and sealed in an evacuated quartz tube that was heated at 1073 K for five days. On cooling to room temperature the compound formed was pulverized and mixed with additional carbon powder (about 20%) before being pressed into a pellet that was annealed using a similar procedure. Ni$_2$MnGa was synthesized by repeatedly arc melting along with intermittent flipping stoichiometric amounts of high purity (> 99.99%) Ni, Mn and Ga in an argon atmosphere. The two resulting samples were powdered and characterized by X-ray diffraction to ensure their formation. Apart from minor impurity phases of MnO and graphitic carbon in Mn$_3$GaC, no other impurities were detected in the two compounds. The elemental compositions of the two compounds were verified using SEM-EDAX. Carbon content was estimated via CHN analysis. The actual compositions of the two compounds were found to be, Mn$_{3.05}$Ga$_{0.98}$C$_{0.97}$ and Ni$_{2.12}$Mn$_{0.92}$Ga$_{0.96}$. Next, the two powders were mixed in the desired proportion, pelletized and encapsulated in an evacuated quartz tube, annealed at 750 $^\circ$C for 48 h and cooled in the furnace to room temperature. X-ray diffraction patterns of these mixtures were recorded at room temperature using Cu K$_\alpha$ radiation in the angular range of 20$^\circ$ to 80$^\circ$ to obtain the structural information. The diffraction patterns were Rietveld refined using the FULLPROF suite software package^27^. A total of 17 parameters which include the background parameters, scale factors and the lattice parameters of the individual phases were refined for the fitting. The composite mixtures were also checked by scanning electron microscopy (SEM) and SEM-EDX for morphology and phase composition. Magnetization measurements, as a function of temperature and magnetic field, were performed on MPMS-SQUID magnetometer. M v T data was recorded in a field of 0.01 T, 1 T, 5 T and 10 T in the temperature interval of 5–400 K. Initially, the samples were cooled in zero applied field down to 5 K, desired field was applied, and the data was recorded while warming (ZFC) as well as during subsequent cooling (FCC) and warming (FCW) cycles. Isothermal magnetization was recorded in magnetic fields up to ± 14 T at 5 K by cooling the sample to the desired temperature in zero applied field (ZFC-MH) as well as in a field of 5 T (FC-MH).
